# A theoretically informed interview study of strategic stakeholders on their readiness to implement a pharmacist competency framework for hospital practice

**DOI:** 10.1007/s11096-026-02101-7

**Published:** 2026-04-02

**Authors:** J. T. Stoll, B. Böhmdorfer-McNair, M. Lutters, A. E. Weidmann

**Affiliations:** 1 Department of Clinical Pharmacy, Innsbruck University, Innrain 52a, 6020 Innsbruck, Austria; 2 Pharmacy Department, Clinic Hietzing, Vienna Healthcare Group, Wolkersbergenstraße 1, 1130 Vienna, Austria; 3 Karl Landsteiner Institute for Clinical Risk Management, 1130 Vienna, Austria; 4 Hospital Pharmacy, Cantonal Hospital Aarau, Tellstraße 25, 5001 Aarau, Switzerland

**Keywords:** Competency framework, Facilitators and barriers, Implementation, Role profile extension

## Abstract

**Introduction:**

Introducing a pharmacist competency framework into a healthcare system has the potential to improve patient care but also poses several challenges. Assessing the readiness of all healthcare stakeholders in position of policy influence is an essential step to identify the correct implementation approach. In Austria, efforts to implement a competency framework for hospital pharmacists are ongoing, yet empirical evidence on the readiness of key healthcare stakeholders remains limited.

**Aim:**

The aim of this study was to explore the readiness of key healthcare policy stakeholders on the possible implementation of a hospital pharmacist competency framework into Austrian hospital practice.

**Method:**

A qualitative interview study underpinned by the Consolidated Framework for Implementation Research (CFIR) with 20 key healthcare policy stakeholders was conducted across Austria. A topic guide and additional study material were developed based on the CFIR. Interview questions were validated and piloted. Interviews were audio-recorded, transcribed ad verbatim and coded in accordance with the CFIR by two researchers independently. Transcripts were analysed using thematic analysis until saturation of themes.

**Results:**

Facilitators and barriers emerged across all five CFIR domains. Key facilitators were aspects of innovation, inner setting and implementation (e.g., relief for physicians due to reducing their workload, teamwork and communication to support implementation, etc.) while key barriers were also related to inner and outer setting, as well as the implementation, specifically the need for adequate structural and financial resources and a sensitive implementation process to not interrupt well-established workflow processes.

**Conclusion:**

This theory-informed study has highlighted the positive attitudes of participants stating their general readiness for implementation. Careful implementation will be necessary not to overwhelm the healthcare system by a sudden change in working structures, processes and hierarchy. These results have impacted policy and educational change for hospital pharmacists across Austria.

**Supplementary Information:**

The online version contains supplementary material available at 10.1007/s11096-026-02101-7.

## Impact statements

- Identified barriers and facilitators by the CFIR will inform the effective and sustainable implementation of the bespoke national clinical pharmacy competency framework for hospital pharmacists into Austrian hospital practice
- Based on this competency framework and the findings of this interview study a healthcare law change was passed, which is now known as the new Delegation Act for Austrian hospital pharmacists
- The competency framework will inform future educational adaptations of the under- and postgraduate education of pharmacists

## Introduction

Global health priorities have increasingly focused on enhancing patient safety and sustainable goals such as ensuring medication without harm [1, 2]. Hospital pharmacists have the possibility to support these priorities with their expertise in optimising medication therapy, reducing medication errors, and contributing to interdisciplinary patient care [3]. They play a critical role in addressing these healthcare challenges, particularly through their ability to collaborate with other healthcare professionals to deliver high-quality, patient-centered care. However, to meet these global demands effectively, hospital pharmacists require clearly defined skills and competencies that support their role in clinical practice [4].

To ensure that hospital pharmacists are equipped with the necessary skills and knowledge, many countries have implemented competency frameworks [4–7]. These frameworks are essential tools in structuring, assessing, and developing the capabilities of healthcare professionals in line with national and international health priorities [4, 5]. While several European countries like the UK or Ireland have established national competency frameworks aligning with broader European standards, many more struggle to implement standardised tools such as the Common Training Framework (CTF) for hospital pharmacists, including Austria [5].

Austria has a traditional hierarchical healthcare system which may limit the flexibility needed to adapt the role of hospital pharmacists in line with international standards [8]. In Austria only 15.8% of the hospitals (n = 266) have their own hospital pharmacy (n = 42) [9]. Clinical pharmacy services in Austria mainly emerged over the past 20 years and are nowadays an essential part of hospital pharmacists’ competencies [10]. By introducing a clinical pharmacy competency framework for hospital pharmacists that addresses Austria’s unique healthcare structure, this study provides essential insights into the readiness of key stakeholders in strategic positions of policy influence. It determines their views on the correct implementation approach, its potential to support hospital pharmacists’ role expansion and any threats by adding complexity to established workflows. These theoretically informed implementation insights may be of relevance for other small, hierarchical healthcare systems.

### Aim

This study aimed to explore the readiness of key Austrian healthcare policy stakeholders on the implementation of a bespoke national clinical pharmacy competency framework into hospital practice, to determine whether the competency framework is seen as a relief or burden for the healthcare system and to identify any associated barriers and facilitators on a national scale to support the implementation into policy and practice.

## Method

### Study design

A qualitative, descriptive approach using semi-structured face-to-face interviews was used. Semi-structured interviews were chosen over focus groups to collect in-depth views and maximise the insight from the different positions of policy influence. The Consolidated Framework for Implementation Research (CFIR) was used to underpin the development of the semi-structured interview questions [11].

### Participant inclusion and exclusion criteria

Key stakeholders were chosen to get a broad overview of all healthcare sectors in Austria (Table 1). They held important positions within their respective healthcare policy related organisations (e.g., president/vice-president) with two stakeholders from each organisation included.

**Table 1:** Healthcare policy related organisations from which participants were chosen

Included organisations (n = 11)
Politics/Ministry of Health
Nursing
Patient Advocacy
Austrian Chamber of Physicians
Tirol Kliniken (University Hospital of Innsbruck)
Austrian Chamber of Pharmacists
Austrian Association of Hospital Pharmacists (AAHP)
Austrian Patient Safety Platform (Plattform Patientensicherheit)
Health Austria GmbH (Gesundheit Österreich GmbH - GOEG)
Austrian Health Insurance Fund (Österreichische Gesundheitskasse - ÖGK)
Universities

### Sampling

Purposive sampling was used in order to include participants. To ensure representation across all included organisations, a target sample size of 22 participants was aimed at. Sandelowski (1995) stated that qualitative sample sizes should be balanced. Large enough to get new and meaningful understanding, yet small enough to allow deep and case-orientated analysis [12]. The sample size (n = 22) was therefore considered sufficient to provide a comprehensive understanding of stakeholder readiness while allowing for in-depth analysis.

**Fig. 1:**
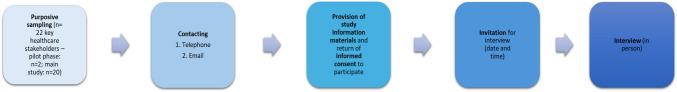
Sampling approach graphically displayed

Initially the names and contact details of possible participants (n = 22) were collated from the information available in the public domain, following which the research team leader (AEW) contacted them via phone, inviting them to participate. If they expressed their interest, they received an email (by JTS) with comprehensive study information (foreword, study information sheet, consent form and a form about their demographic background). If they decided to participate in the study, they had to sign the consent form, fill out the form about their demographic background and sent these forms back (to JTS). Following this, an interview date was agreed (by JTS) which was most suitable for the participants (see Fig. 1). Finally, 22 key stakeholders were included, whereas two (10% of the sample size) were included for the pilot phase. Therefore, 20 participants were included in the main interview study.

### Development of study materials and pilot phase

Based on best practice papers and already published literature the study materials were developed [13–16]. Two researchers (JTS/AEW) developed the interview topic guide, which was based on the existing literature, as well as contextual circumstances and the Consolidated Framework for Implementation Research (CFIR) [11, 15, 16]. The CFIR consists of five domains and an additional outcomes domain. An overview of the topic guide and the included domains is provided in Table 2.

**Table 2:** Topic guide on the possible implementation of a clinical pharmaceutical competency framework for Austria and the CFIR domains used

Question number	Main question	Sub question/explanation	CFIR domain
*Welcoming*	*My name is Jxxxxx Sxxxx. Today I am conducting an interview with you on the possible implementation of a bespoke national clinical pharmacy competency framework in Austria. I will ask you a few questions about the necessity, legal aspects, implementation and your personal opinion. Thank you again for your participation in the study.*	*Are there any questions in advance?* *If yes, answer questions.* *If not, start the interview.* *The recording will then start now.*	
*Knowledge elicitation*			
1	Are you familiar with the term ‘Clinical Pharmacy Competency Framework’?	If yes, what do you mean by this? If no, explanation. Or if incorrect, explanation. The practical skills that a pharmacist must have in order to optimally contribute to the patient’s therapy in an interdisciplinary team. These skills are summarised in a document and legally clarified.	1 (Innovation)
2	Have you ever had experience with such a competency framework outside of Austria?	If yes, which one and in which role? If no, continue.	4 (Individuals)
*Necessity*			
3	Do you think it is necessary to define competencies for pharmacists in Austria for their field of activity in hospitals?	Can you please explain this in more detail?	1 (Innovation)
4	What do you think could be the advantages/disadvantages of such a competency framework (for patients, healthcare professionals or your organisation)?	Who do you think would benefit most from such a competency framework?	1 (Innovation)
5	Do you think patient safety in Austria could be improved with a clinical pharmacy competency framework?		1 (Innovation)
*Law*			
6	Do you think hospital pharmacists should be granted an extension of their statutory rights with regard to patient-centred therapy?	If yes, which one? If no, why?	2 (Outer Setting)
7	What changes or adjustments do you think would be necessary to implement such a competency framework in Austria?	Explanation: Changes in the healthcare system, in the field of activity of hospital pharmacists, teaching/training and laws. Who do you think should get something like this rolling?	3 (Inner Setting)
*Implementation*			
8	What resources would be needed to successfully implement a clinical pharmacy competency framework in Austria?	How could these resources be procured? What difficulties could arise in obtaining resources? What do you think is the biggest obstacle to obtaining resources? How do you think this can be overcome? What processes would make obtaining resources easier?	3 (Inner Setting)
9	In what time frame could a possible implementation of the competency framework take place?		3 (Inner Setting)
*Personal*			
10	In your opinion, how can you contribute to the implementation of a clinical pharmacy competency framework?		4 (Individuals)
11	Would you be willing to make resources available for the implementation of the competency framework?	If yes, which resources? If not, why not?	4 (Individuals)
12	Do you have any further comments on the competency framework (which we have not yet discussed)?	If no, end the interview.	4 (Individuals)
*Ending the conversation*	*Thank you very much for your participation. If you have any further questions, please do not hesitate to contact us. Otherwise, this concludes the interview.*	*Stop recording.*	

The CFIR was chosen to theoretically inform this interview study as it is a meta-theoretical framework synthesising multiple pre-existing implementation theories and models. Face and content validity were obtained by review of the study materials by two experienced researchers (DM/DD) independent to the study. For the pilot phase two key stakeholders (one each from the academic sector and the Austrian Association of Hospital Pharmacists) were interviewed. Interviews from the pilot study were not included in the final dataset, as small corrections to the questions, such as the deletion of sub questions due to a lack of additional information returned was necessary.

### Data generation and storage

Interviews were conducted between June and October 2023 at the participants workplace (e.g., Ministry of Health, hospital pharmacy, etc.) and lasted up to 45min. Before the interview, informed consent was re-confirmed, participants were reminded that the interview will be recorded and that they can withdraw from the study at any point without providing justification. Participants were offered to receive the transcript of their interview after transcribing to check if they feel represented correctly. During the interview, field notes were taken by the interviewer (JTS) if necessary. An audio device was used for recording and the audio file transferred to a password-protected file location on a secure University of Innsbruck server. The audio files were transcribed ad verbatim (JTS) using Microsoft Word (vs. 2021). Prior to analysis all personal details of the participants were pseudonymised.

### Data saturation

In addition to the a priori sampling goal (n = 22), the sample size determination of Francis et al. (2010) was used to identify the point of data saturation [17]. The research team agreed on a stopping criterion of three (three more interviews in case no new data emerge).

### Data analysis

#### Coding

The qualitative coding software MAXQDA (vs. 24.4.0) was used. All transcripts were uploaded and the constructs of the CFIR were used to form a coding framework. Coding was conducted by four researchers independently (JTS/BBM/ML/AEW), whereas JTS coded all transcripts and compared them with the codes of the other researchers. In case of discrepancies these were resolved through discussion and if no agreement could be reached a third researcher was consulted (BBM/ML/AEW). Consistency of the codes was checked throughout (JTS).

### Thematic analysis

After extensively familiarising herself with the data, themes were created by JTS. They were independently reviewed (AEW) and discussed in case of uncertainties. Themes were also reviewed by two other researchers independently (BBM/ML). Interviews and analysis were conducted in the German language. Therefore, the representative quotes in this paper had to be translated by JTS and were reverse translated by a bilingual native English speaker (AEW). A third researcher was consulted in case of discrepancies during translation to reinforce trustworthiness.

To ensure consistency throughout, the identified themes were mapped again to the associated CFIR constructs (JTS) in a following step. Facilitators and barriers were identified (JTS) within these themes and associated constructs, and were summarised according to the CFIR domains. This process was independently checked by another researcher (AEW). In case of discrepancies, these were discussed and if no agreement could be reached, a third researcher was consulted (BBM/ML). The impact of the researchers own background as a pharmacist was considered minimal as she had no prior experience as a hospital pharmacist and no prior relationship to any of the stakeholders interviewed.

### Ethics approval

As no patient sensitive information was included in this study, the Ethics Committee of the Medical University of Innsbruck stated on the 14^th^ of December 2022 that an ethical approval was not necessary. Nevertheless, the study was conducted according to the good scientific practice rules stated by the World Medical Association (WMA) in the Declaration of Helsinki, the Guidelines for Good Scientific Practice by the Austrian Agency for Research Integrity, and the internal scientific practice rules of the University of Innsbruck [18–20].

## Results

### Demographic background

The demographic background of participants is shown in Table 3. Their workplaces were distributed across Austria, although it was not possible to recruit participants from each federal state. Their specific, self-reported level of experience with clinical hospital pharmacy services ranged from ‘a lot’ (n = 11), to ‘some’ (n = 6), and ‘none’ (n = 3).

**Table 3:** Summary of the demographic background of participants

Participant number	Organisation	Federal state	Level of experience with clinical hospital pharmacy services
1	Austrian Chamber of Physicians	Salzburg	A lot
2	Austrian Chamber of Physicians	Tyrol	None
3	Nursing	Tyrol	Some
4	Tirol Kliniken (University Hospital of Innsbruck)	Tyrol	A lot
5	Patient Advocacy	Lower Austria	A lot
6	Health Austria GmbH (Gesundheit Österreich GmbH - GOEG)	Vienna	Some
7	Politics/Ministry of Health	Vienna	Some
8	Patient Safety Platform (Plattform Patientensicherheit)	Vienna	A lot
9	Universities	Salzburg	A lot
10	Austrian Health Insurance Fund (Österreichische Gesundheitskasse - ÖGK)	Tyrol	None
11	Universities	Styria	Some
12	Austrian Chamber of Pharmacists	Vienna	A lot
13	Health Austria GmbH (Gesundheit Österreich GmbH - GOEG)	Vienna	Some
14	Patient Safety Platform (Plattform Patientensicherheit)	Vienna	A lot
15	Austrian Association of Hospital Pharmacists (AAHP)	Lower Austria	A lot
16	Patient Advocacy	Tyrol	Some
17	Austrian Association of Hospital Pharmacists (AAHP)	Tyrol	A lot
18	Tirol Kliniken (University Hospital of Innsbruck)	Tyrol	A lot
19	Austrian Chamber of Pharmacists	Vienna	A lot
20	Politics/Ministry of Health	Tyrol	None

### Thematic analysis

**Fig. 2:**
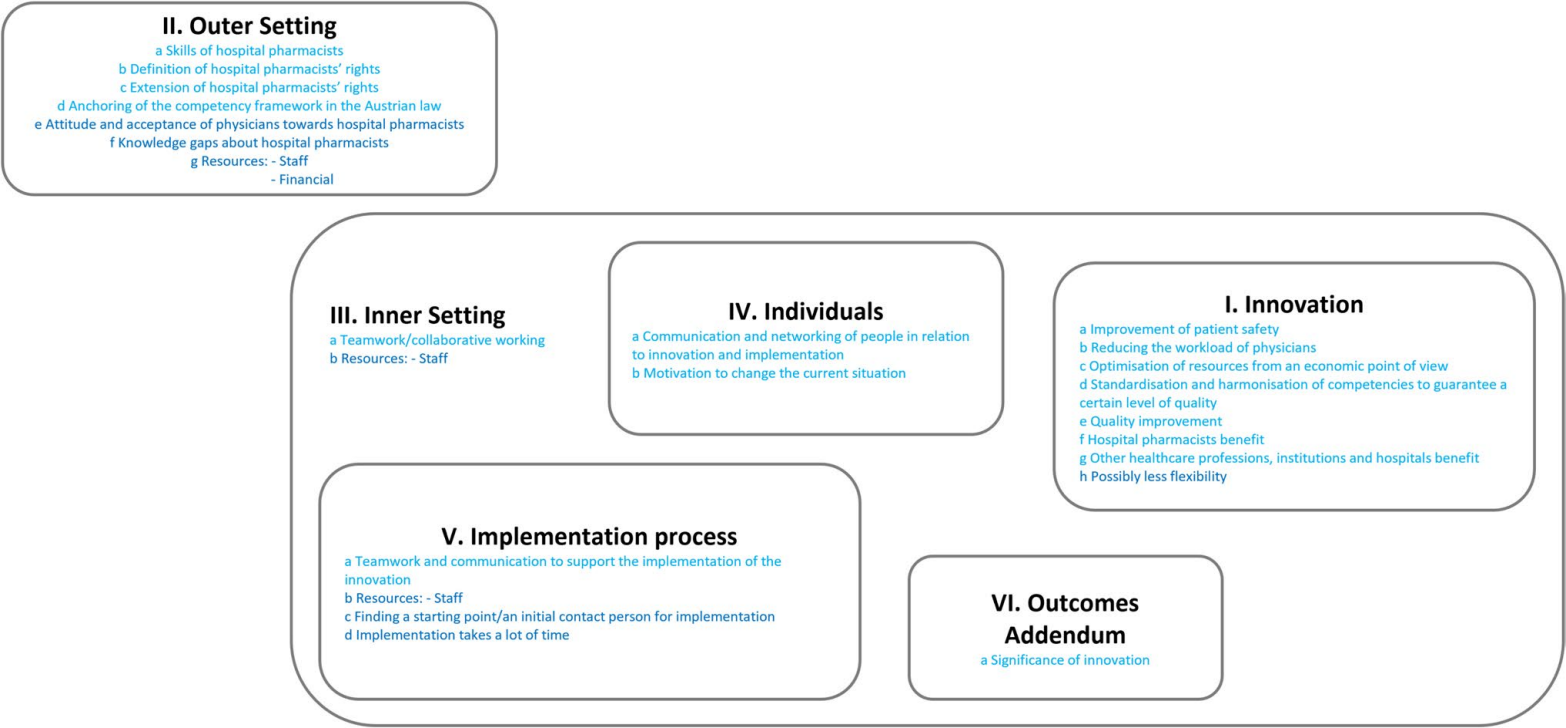
This figure shows a summary of facilitators and barriers structured by CFIR domains; Light blue: Facilitators/positive factors for implementation; Dark blue: Barriers/negative factors for implementation

Identified themes reflect both benefits (relief) and challenges (burden). Overall, the framework was largely seen as positive (see Innovation domain, Fig. 2) for the healthcare system, but participants were concerned about how to best conduct the implementation process. All identified themes, associated constructs and related facilitators/barriers are provided in the supplementary material (Supplementary Table 1).

### Facilitators (Benefits)

#### Relief for the healthcare system (CFIR domain innovation)

Some participants felt that the healthcare system has reached its limit and expressed their wish for someone who could support them regarding medication therapy (see Fig. 2; Innovation a, b, c, e, g).

"Our institution has clearly identified, that we … need someone ... because there are … more and more new drugs." (IP4, Medical Director)

"If you do your job [hospital pharmacist] well … and ensure the ... rational pharmacoeconomic use of medicines, ... you will definitely recoup your costs." (IP9, University Professor of Pharmacy)

#### Relief for physicians (CFIR Domain Innovation)

Physicians highlighted that they would be in favour to have a hospital pharmacist by their side, although other participants believed that not all physicians would see the advantages of that right away (see Fig. 2; Innovation e, g).

"... it would relieve us [physicians] of a lot of ... stress and would be an absolute benefit for… quality improvement and patient safety. So, I am absolutely in favour of it." (IP4, Medical Director)

"... [the competency framework] brings relief [in relation to the burden of the physicians] ... But that would probably be something that physicians wouldn’t ... see straight away …, and there’s probably also ... a bit ... of a generational thing, that a younger generation ... is more willing than ... grey eminences ..." (IP13, Pharmaceutical Economist)

#### Patient outcomes (CFIR Domain Innovation)

It was believed that patient outcomes such as mortality (see Fig. 2; Innovation a) will improve with the help of hospital pharmacists*.*

"...interactions are becoming more and more unclear ... and I think that a lot ... of morbidity, mortality ... could ... be reduced or ... influenced .... So, I’m convinced that it would be good, yes ..." (IP4, Medical Director)

#### Abilities of hospital pharmacists and their future professional development (CFIR Domain Outer Setting)

Skills and knowledge of hospital pharmacists (see Fig. 2; Outer Setting a) were already known to some participants and they believed that the competency framework will help to define the hospital pharmacist’s role in the interdisciplinary team (see Fig. 2; Outer Setting b, c, d). Furthermore, they viewed the competency framework as a basis for advancing the hospital pharmacists profession.

"... I can already get expertise from the non-medical field (e.g., hygiene) and I can gain additional expertise from the [Clinical Pharmacy]." (IP2, Physician in a political position)

"... Such a competency framework could, … identify very clearly the boundaries of responsibility for one professional group [compared to others]…" (IP5, Patient Advocate)

"I would say that the establishment of a competency framework is the essential prerequisite for the further development of our profession." (IP17, Hospital Pharmacist)

#### Barriers (Challenges)

#### System readiness (CFIR Outcomes Addendum)

Participants stated that the more prepared the system is, the easier it will be to implement the competency framework (see Fig. 2; Outcomes Addendum a).

"... I don’t know if we’re that far yet. ... it raises the question whether it has to be such an abrupt change or whether it should be introduced gradually so that the ... systems, such as hospitals or surgeries etc., ... get used to it." (IP16, Patient Advocate)

#### Concerns about change (CFIR Domains Innovation and Outer Setting)

Concern was expressed about how the implementation of the competency framework might affect current workflow processes if it is not flexible enough to be adaptable to specific needs (see Fig. 2; Innovation h).

"I could imagine that it [competency framework] ... would be too rigid a scheme. That ...things might be adopted in a very generalised way when it should almost certainly be individualised ..." (IP4, Medical Director)

Some physicians feared task encroachment by hospital pharmacists. Defining the role of hospital pharmacists through a competency framework might therefore help to reduce their skeptical attitude towards hospital pharmacists (see Fig. 2; Outer Setting b, c, d, e).

"... Physicians are at the top of the healthcare pyramid and if someone else is possibly at the same level ..., then that is somehow perceived as downgrading the medical profession." (IP5, Patient Advocate)

"It is also about expanding the current competencies ..., which might require a legal change and I wonder ... will other professional groups see this as competition? Will they ... be against a competency framework because they have the sense that you [hospital pharmacists] ... are encroaching on their territory?" (IP13, Pharmaceutical Economist)

#### Law changes (CFIR domain outer setting)

Legal changes would be necessary in order to use the competency framework to a full extent (see Fig. 2; Outer Setting b, c, d). Some participants favoured standardisation of laws for Austrian hospital pharmacists, whereas others aimed for expanding rights and a new delegation law.

"Because it’s ... important, especially in such a federal country like Austria ... to proceed in a standardised way,..." (IP19, Pharmacist in a political position)

"... we have already ... spoken to the Federal Ministry and ... the Pharmacy Chamber and ... are endeavour to ensure that paragraph 36 of the Pharmacy Act includes a role expansion for pharmacists in hospitals working under the delegation of a medical prescriber ..." (IP17, Hospital Pharmacist)

#### Lack of resources (CFIR domains outer and inner setting)

Participants were unsure who will pay for hospital pharmacists services (see Fig. 2; Outer Setting g).

"It won’t work without resources ... when money is involved, there is always ... a long discussion about the allocation to be had." (IP6, Health Expert in Pharmacoeconomics)

Other countries have already realised the importance of hospital pharmacists long time ago due to problems in the healthcare system (e.g., staff shortages; see Fig. 2; Inner Setting b). As Austria is recently facing a similar situation, this might be an important factor when it comes to implementation.

"... 20 years ago in England, ... there was a shortage of physicians … and ... there was a sudden realisation that there is a professional group that really knows a lot about medicines ... so they [pharmacists] were integrated into the process. That has obviously ... not yet been as necessary ... in the existing [Austrian] system." (IP17, Hospital Pharmacist)

Problems like skilled staff shortages need to be overcome and require a change in the healthcare system (see Fig. 2; Outer Setting g).

"... with the current skilled staff shortage, ... perhaps the real question we should ask ourselves is ... while we want this support of hospital pharmacists ..., we may wonder where will we get it from, in five years’ time? ... So, anyone who ignores this problem is making a huge mistake." (IP18, Medical Director)

#### Partnering and cooperation (CFIR domains individuals and implementation process)

Participants agreed that finding the right cooperation partners is a crucial step in the implementation process, although it might be difficult to find the right partners (see Fig. 2; Individuals a; Implementation process a). They were also unsure where and with whom to start the implementation process (see Fig. 2; Implementation process c).

"Politicians probably have to support it [the implementation process] and the respective professional bodies ... Not just the Chamber of Pharmacists, but also the medical and nursing profession." (IP9, University Professor in Pharmacy)

"... you have to have formal talks ... with the people in charge, that means specifically ... in the hospital system you must have the Ministry of Health ... on board ... . … you have to formally get them on board ..., so not just impose it on the whole of Austria, but look for allies ... and take the first steps ... right up to the health policy level.” (IP19, Pharmacist in a political position)

"I believe that ... we [federal state governance] are not the right contact person ...." (IP20, Politician)

"... we are finding it a little difficult as a federal government [country level] to take this forward as a major initiative ... that is so strongly anchored within the governance level of the federal state ... ." (IP7, Politician)

#### Facilitators and barriers regarding competency framework implementation revealed by framework analysis (CFIR)

See fig 2

## Discussion

The findings of this study highlighted the perceived readiness and the practical uncertainties that need to be considered for successful implementation. Participants were aware of the numerous advantages hospital pharmacists have on the healthcare system (e.g., reduced workload of physicians, economical aspects, etc.) and believed that a competency framework would help to underpin and define their skills and roles within the healthcare system. While participants seemed ready for implementation, they were unsure about how to conduct the actual implementation process as concerns over the disturbance of established workflows and healthcare hierarchies existed.

Participants positive perceptions of hospital pharmacists’ contribution align with the findings of other studies demonstrating their role in improved patient outcomes, reduced workload for other healthcare professionals, and lower healthcare system costs [21, 22]. Defining hospital pharmacists’ competencies will also help to reshape education and further training, as it is known on what the curriculum should focus on [23, 24]. While Austria provides Bologna-aligned undergraduate programmes in pharmacy, available postgraduate hospital pharmacy training opportunities remain sparse with one centrally organised, legally bound specialisation programme available [10, 25]. The previously developed bespoke national clinical pharmacy competency framework could be used to update the teaching of the hospital pharmacist degree [26]. Challenges in the healthcare system such as skilled staff shortages, need to be overcome to maintain patient safety at the highest possible level [27, 28]. Starting in the 1970s, clinical pharmacy services provided by hospital pharmacists in the UK evolved [29, 30]. Showing how much they can contribute to patient care, health economics, and supporting interdisciplinary healthcare teams, their role got a more interdisciplinary focus [29, 30]. In 2019, the new general practitioner (GP) five-year contract framework was published in the UK, which allowed Primary Care Networks (PCN) to recruit up to 20,000 additional staff, including clinical pharmacists [31]. Comparing this process in the UK to the development of the hospital pharmacist profession in Austria, there is much more that could be done to support the healthcare system and especially patients’ safety.

One driver for change identified in this interview study was participants dissatisfaction with the current work pressures in hospitals. Some physicians expressed that they are overwhelmed with the number of new drugs and interactions and their need for additional expert support to concentrate on other urgent tasks such as diagnostics and acute patient care. This is in line with the findings of a study by Elliott et al. (2023) where physicians welcome the support of hospital pharmacists as they help to reduce their workload, work-related stress and burnout levels [22]. Hatton et al. have shown that hierarchical structures are hindering the collaboration in interdisciplinary teams which was similarly reflected in this study [32]. Implementation however was seen as a crucial and multi-step approach that needs to be conducted considering existing workflows and structures, allowing time for gradual adaptation. Successful and sustainable implementation of new practices or services requires behaviour change which can be challenging, as it means to break old behaviour patterns and convince individuals that new methods can be better [32, 33]. Therefore, implementation has to be done carefully to not interrupt existing workflow systems which was also supported by the participants [34, 35]. Including stakeholders can facilitate implementation but can also be challenging in case of conflicting interests or an unclear direction for implementation [34]. Participants acknowledged the need to involve all key stakeholders in the implementation process, yet struggled to identify appropriate implementation partners, likely due to the multi-level structure of the Austrian healthcare system [8].

The advantages of defining hospital pharmacists’ role profile and the wish especially from physicians for implementation were used to inform a legal role extension for hospital pharmacists in Austria. In July 2024, a new Delegation Act for hospital pharmacists was passed, supported by the results of this study, as they were used to facilitate the discussions of the Austrian Association of Hospital Pharmacists (AAHP) and the legal department of the Austrian Chamber of Pharmacists with the former Austrian health minister [36]. This new Delegation Act, allows hospital pharmacists to take on certain tasks under the delegation of a medical prescriber. This includes: replacement of a prescribed medication; adjustment of the pharmaceutical form, quantity and strength of the prescribed medication; termination, continuation or interruption of drug therapy [36]. While Austrian hospital pharmacists are thrilled about the first law change since 1984, they are concerned about having the required competencies. This is likely due to the existing traditional and pharmaceutical science related, rather than being more focused on interprofessional and patient-care practice as dictated by the EU curriculum [37, 38]. Similar issues arose in the UK, after pharmacists received the legal right to prescribe independently [39]. Despite their extensive training and competencies, pharmacists were reluctant to accept this responsibility due to concerns that a prescribing mistake by a pharmacist will get handled differently to that of a medical prescriber [40, 41]. Using the new bespoke competency framework as the basis for pharmacists’ education and further training going forward, will help to upskill Austrian hospital pharmacists and empower their belief in their own competencies [26]. For Austria, these findings could be used next to inform a structured extension of the hospital pharmacists’ role profile codex (ABO), thereby facilitating the gradual implementation of the bespoke national competency framework into hospital practice [42].

### Strengths & limitations

The study was developed based on other similar studies and underpinning research frameworks (e.g., CFIR) were used throughout to enhance credibility and dependability and to support transferability. Participants were ensured confidentiality, they were encouraged to answer honestly and it was aimed to keep bias (e.g. social desirability bias) to a minimum, which supports the confirmability of this study. Despite concerns over a representational imbalance by only interviewing senior executives, no evidence that the power dynamics meaningfully constrained the data was observed. The interviewer (JTS; PhD student) received training on how to conduct interviews appropriately. It was not possible to recruit a participant of each federal state. Interviews and analysis were conducted in the German language. Linguistic minutia might have been lost in the reverse translation process. However, the meaning of participants’ statements was preserved, and the findings remain transferable to similar healthcare contexts. Moreover, it was not possible to reach data saturation which was due to the heterogenous background of participants.

## Conclusion

Key healthcare stakeholders’ readiness for implementation and a careful implementation process could be drivers for change within Austria. Key facilitators such as advantages of the innovation, including the relief for the healthcare system and physicians, as well as teamwork and communication could support implementation, while key barriers including limited financial and structural resources have to be considered. Beyond the Austrian context, this study offers valuable insights for other European countries without competency frameworks struggling with the role profile development of hospital pharmacists. Successfully extending pharmacists’ roles depends not only on legal recognition but also on education and further training that can guide future healthcare reforms across Europe. It is essential that policymakers and professional bodies collaboratively adapt hospital pharmacists’ role profiles, allocate necessary resources, and provide targeted training to ensure effective and sustainable implementation of competency frameworks in practice.

## Supplementary Information

Below is the link to the electronic supplementary material.

Supplementary file 1 (DOCX 25 KB)

## Data Availability

Data is not available to third parties due to participants not agreeing that their data will be shared.
