# Supplementary material for: A theoretically informed interview study of strategic stakeholders on their readiness to implement a pharmacist competency framework for hospital practice
Source: Int J Clin Pharm. 2026 Apr 2;48(3):1036–47. doi: 10.1007/s11096-026-02101-7 (PMC13176179; doi:10.1007/s11096-026-02101-7)
Supplement: Supplementary file 1 — (DOCX 25 KB) [file 11096_2026_2101_MOESM1_ESM.docx]

**Supplementary material**

| Supplementary Table 1 Themes identified in this interview study with their associated CFIR constructs and related facilitators and barriers structured by the CFIR domains (NA= not applicable) | | | |
| --- | --- | --- | --- |
| CFIR domain | **Themes** | **Associated constructs** | **Facilitators/Barriers** |
| Innovation | Advantages of the innovation | C. Innovation Relative Advantage | I. Innovation a, b, c, d, e, g |
|  | Usability of the competency framework in practice | B. Innovation Evidence-Base  D. Innovation Adaptability | I. Innovation f  I. Innovation h |
|  | Pilot studies | E. Innovation Trialability | NA |
| Outer Setting | Skills & knowledge of hospital pharmacists | C. Local Conditions | II. Outer Setting a |
|  | Legal changes | E. Policies & Laws | II. Outer Setting b, c, d |
|  | Competition between healthcare professionals | B. Local Attitudes  C. Local Conditions | II. Outer Setting e |
|  | Healthcare system | B. Local Attitudes  C. Local Conditions | II. Outer Setting f, g  I. Innovation a, d, e |
|  | Different local circumstances | B. Local Attitudes  C. Local Conditions  F. Financing | II. Outer Setting g |
|  | Resources | C. Local Conditions  F. Financing | II. Outer Setting g  I. Innovation c |
|  | Media | G. External Pressure, 1. Societal Pressure | NA |
|  | Goals of the competency framework implementation | G. External Pressure, 2. Market Pressure, 3. Performance-Measurement Pressure | II. Outer Setting b, d |
| Inner Setting | Working on ward | A. Structural Characteristics, 2. Information Technology Infrastructure, 3. Work Infrastructure  B. Relational Connections | III. Inner Setting a |
|  | Cultural aspects | D. Culture | NA |
|  | Urgency and necessity of the implementation | A. Structural Characteristics, 3. Work Infrastructure  C. Tension for Change | III. Inner Setting b |
|  | Professional development of hospital pharmacists | I. Mission Alignment | NA |
| Individuals | Knowledge & Experience | A. Need  B. Capability  C. Opportunity | IV. Individuals a |
|  | Motivation for change | D. Motivation | IV. Individuals b |
| Implementation Process | Partnering & cooperation | A. Teaming  C. Assessing Context  F. Engaging | V. Implementation process a  V. Implementation process c |
|  | Finances | C. Assessing Context | V. Implementation process b |
|  | Status quo | B. Assessing Needs  C. Assessing Context | NA |
|  | Implementation process | D. Planning  E. Tailoring Strategies | V. Implementation process c, d |
|  | Roles in the implementation process | F. Engaging, 1. Innovation Deliverers | NA |
|  | Strategic plans for implementation | E. Tailoring strategies | NA |
|  | Review & Assessment | H. Reflecting & Evaluating | NA |
| Outcomes Addendum | Readiness for Implementation | I. Antecedent Assessments, A. Acceptability, C. Feasibility, E. Implementation Readiness | VI. Outcomes Addendum a |
